# The Incorporation of Marine Coral Microparticles into Collagen-Based Scaffolds Promotes Osteogenesis of Human Mesenchymal Stromal Cells via Calcium Ion Signalling

**DOI:** 10.3390/md18020074

**Published:** 2020-01-23

**Authors:** Eamon J. Sheehy, Mark Lemoine, Declan Clarke, Arlyng Gonzalez Vazquez, Fergal J. O’Brien

**Affiliations:** 1 Tissue Engineering Research Group (TERG), Department of Anatomy and Regenerative Medicine, Royal College of Surgeons in Ireland, D02 YN77 Dublin, Ireland; eamonsheehy@rcsi.ie (E.J.S.); marklemoine@rcsi.ie (M.L.); 2 Trinity Centre for Biomedical Engineering, Trinity Biomedical Sciences Institute, Trinity College Dublin, D02 R590 Dublin, Ireland; 3 Advanced Materials and Bioengineering Research Centre, Royal College of Surgeons in Ireland and Trinity College Dublin, D02 YN77 Dublin, Ireland; 4 Zoan Biomed Ltd., An Luslann, Kylebroughlan, Moycullen, H91 TXV5 Co Galway, Ireland; declanclarke@zoanbiomed.com

**Keywords:** bone, tissue engineering, mechanical properties, calcium, bone morphogenetic protein, alkaline phosphatase, calcium sensing receptor

## Abstract

Composite biomaterial scaffolds consisting of natural polymers and bioceramics may offer an alternative to autologous grafts for applications such as bone repair. Herein, we sought to investigate the possibility of incorporating marine coral microparticles into a collagen-based scaffold, a process which we hypothesised would enhance the mechanical properties of the scaffold as well its capacity to promote osteogenesis of human mesenchymal stromal cells. Cryomilling and sieving were utilised to achieve coral microparticles of mean diameters 14 µm and 64 µm which were separately incorporated into collagen-based slurries and freeze-dried to form porous scaffolds. X-ray diffraction and Fourier transform infrared spectroscopy determined the coral microparticles to be comprised of calcium carbonate whereas collagen/coral composite scaffolds were shown to have a crystalline calcium ethanoate structure. Crosslinked collagen/coral scaffolds demonstrated enhanced compressive properties when compared to collagen only scaffolds and also promoted more robust osteogenic differentiation of mesenchymal stromal cells, as indicated by increased expression of bone morphogenetic protein 2 at the gene level, and enhanced alkaline phosphatase activity and calcium accumulation at the protein level. Only subtle differences were observed when comparing the effect of coral microparticles of different sizes, with improved osteogenesis occurring as a result of calcium ion signalling delivered from collagen/coral composite scaffolds. These scaffolds, fabricated from entirely natural sources, therefore show promise as novel biomaterials for tissue engineering applications such as bone regeneration.

## 1. Introduction

Tissue engineering applications combine cells, biomaterials and signalling factors with the aim of regenerating or replacing damaged tissues [1]. Biomaterials for tissue engineering applications are often presented in the form of three-dimensional (3D) porous scaffolds which can be leveraged as grafts to facilitate host cell infiltration and extracellular matrix (ECM) deposition upon implantation in vivo [2]. Alternatively, scaffolds can act as templates onto which cells, such as mesenchymal stromal cells (MSCs), can be seeded and subsequently primed in vitro to differentiate down tissue-specific lineages, thereby leading to the development of functional engineered tissues [3]. Generally, three types of materials are used in the fabrication of such 3D scaffolds; ceramics, synthetic polymers and natural polymers, each of which offer distinct advantages and disadvantages for use in orthopaedics and tissue engineering applications such as bone regeneration.

Marine coral is a natural bioceramic which has been examined as a graft for bone repair due its inherent porosity and high compressive stiffness [4,5,6,7,8,9]. However, the field of bone tissue engineering is increasingly moving away from the fabrication of such ‘hard’ scaffolds. This is to a large extent due to the brittleness of ceramic scaffolds as well as the difficulty attributed with controlling their degradation rate [10]. The insufficient capacity of such scaffolds to promote vascularisation upon implantation is another major hurdle [11]. Vascularisation of an implanted graft is key to its survival as it ensures the supply of oxygen and nutrients to central regions of the graft, thereby preventing core necrosis and avoiding failure of the construct [12]. To that end, there has been a shift in the field towards the fabrication of highly porous ‘soft’ scaffolds which can preferentially guide the rapid migration of cells and blood vessels into core regions of the scaffold [13]. 

Collagen is an attractive natural polymer for use as a biomaterial as it is the main component of the ECM and offers binding sites for cells, known as ligands, on the its surface [14,15]. Furthermore, collagen can be processed by manufacturing techniques such as freeze-drying in order to fabricate highly porous 3D scaffolds, the porosity of which can be tailored to ensure efficient cell migration into central regions of scaffolds whilst also providing avenues for nutrient diffusion and waste removal [16,17,18]. Collagen scaffolds, however, are hindered by their poor mechanical properties, which may limit their use in orthopaedic applications such as bone repair. In this regard, the incorporation of bioceramics can be advantageous, as they can be utilised in particulate form to reinforce the architectural and mechanical structure of a soft scaffold [19], whilst also potentially acting to deliver pro-osteogenic cues to cells migrating through the scaffold. Indeed, we have previously taken this approach with synthesised hydroxyapatite (HA), whereby the addition of HA microparticles to a collagen-based scaffold was found to enhance its compressive stiffness and permeability [20] and was ultimately shown to promote bone regeneration in vivo [21]. Such a strategy could also potentially be executed using natural bioceramics like marine coral, which are typically comprised of calcium carbonate. It remains unclear, however, the optimum microparticle size that should be utilised when leveraging ceramic particles in this manner, although studies from our group have previously indicated that particle sizes of less than 100 µm are suitable both for promoting osteogenesis and ensuring a reduced immune response [22,23].

The objective of this study was to investigate the possibility of fabricating a novel biomaterial scaffold for bone tissue engineering applications by incorporating marine coral microparticles into a collagen-based scaffold, a process which we hypothesized would enhance the mechanical and osteogenic properties of resultant biomaterial. Coral microparticles of different sizes were achieved by cryomilling and sieving through 100 µm and 45 µm pore sieves. These coral microparticles were then separately incorporated into collagen-based scaffolds and X-ray diffraction (XRD) and Fourier transform infrared (FTIR) spectroscopy was utilised to determine the mineral composition of both the initial coral material and the subsequent collagen/coral composite scaffolds. Thereafter, the effect of microparticle size on the chemical composition, architecture and mechanical properties of collagen/coral scaffolds were evaluated. Finally, we investigated the capacity of collagen/coral scaffolds to promote osteogenesis of human MSCs in vitro with the goal of optimising the development of a highly porous collagen/coral scaffold for use as a novel graft for promoting bone regeneration. 

## 2. Results

### 2.1. Material Characterisation of Coral Microparticles and Collagen/Coral Scaffolds

**Figure 1:**
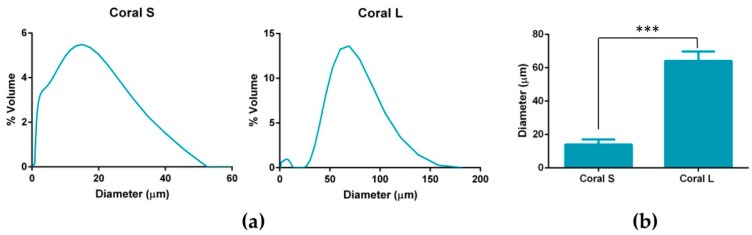
(**a**) Representative volumetric distribution of coral S and coral L microparticles. (**b**) Mean volume weighted diameters of coral S and coral L microparticles. Significance; *** *p* < 0.001 as determined by unpaired t test (*n* = 3).

In order to achieve coral microparticles of different sizes, cryomilled particles were sieved through 100 µm and 45 µm sieves with particles that passed through the 45 µm sieve being designated as small (S) and particles that did not pass through the 45 µm sieve but did pass through the 100 µm sieve being designated as large (L). Dynamic light scattering was used to calculate the volumetric distributions and mean diameters of both microparticle sizes and representative distributions are shown in Figure 1a. The mean diameter of coral L microparticles was found to be significantly higher when compared to that of coral S microparticles (64.11 ± 5.69 µm vs. 13.94 ± 3.14 µm; *p* = 0.0002) (see Figure 1b). 

**Figure 2:**
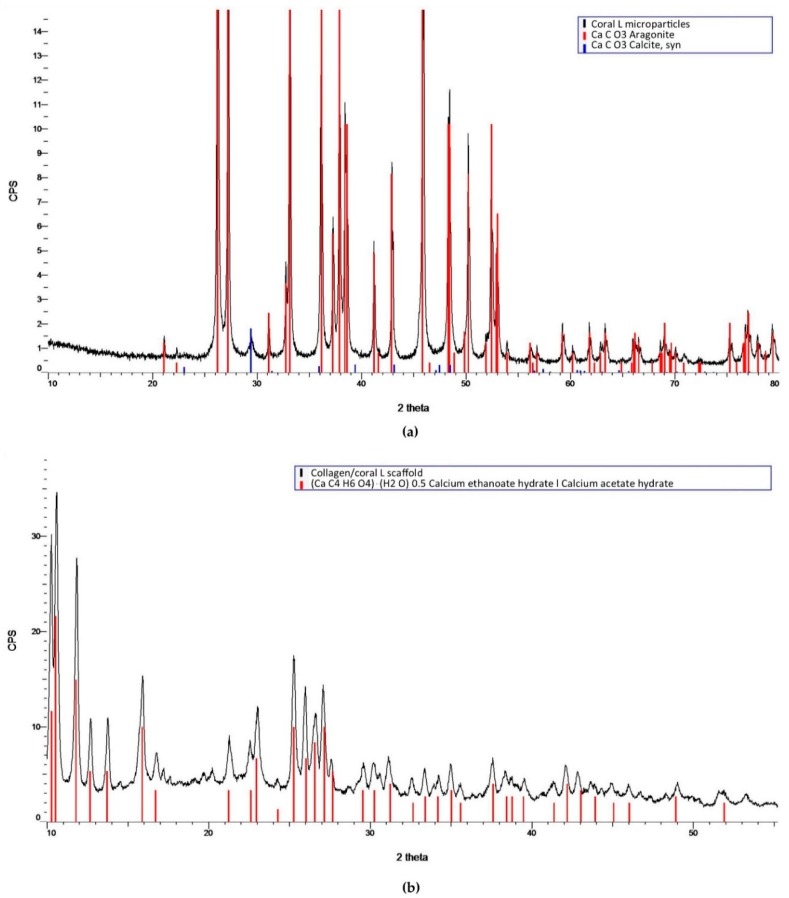
(**a**) XRD analysis of coral large (L) microparticles. (**b**) XRD analysis of collagen/coral L scaffolds. Control spectra were obtained from the International Centre for Diffraction Data (ICDD); CaCO_3_ Aragonite–PDF 00-041-1475 (ICDD, 2019), CaCO_3_ Calcite, syn–PDF 00-005-0586 (ICDD, 2019), (CaC_4_H_6_O_4_)·(H_2_O) 0.5 Calcium ethanoate hydrate׀Calcium acetate hydrate–PDF 00-019-0199 (ICDD, 2019). CPS indicates counts per second.

**Figure 3:**
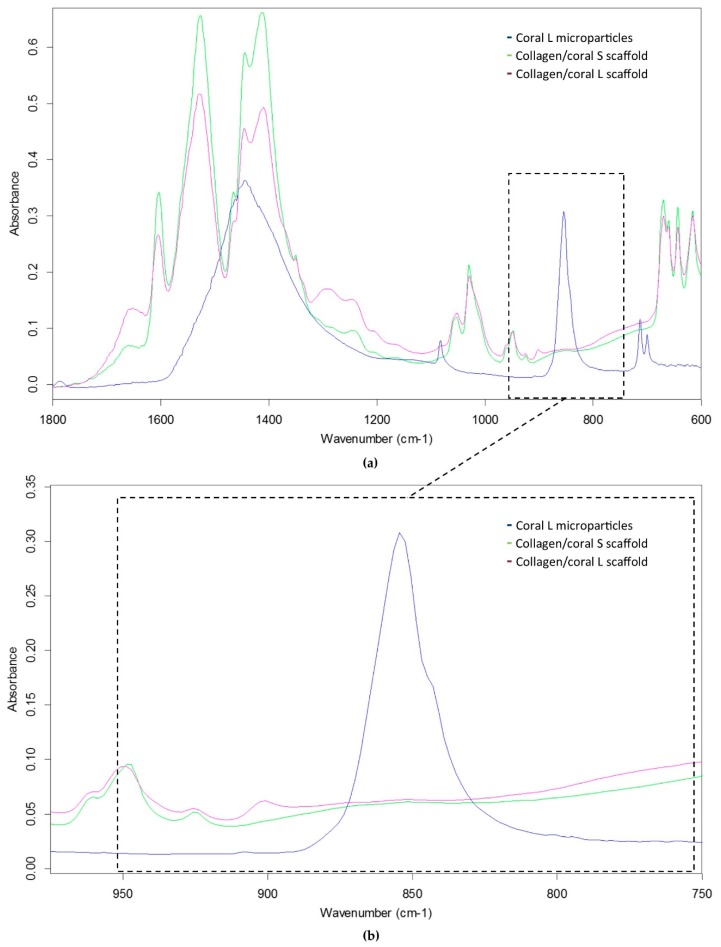
(**a**) FTIR spectroscopy of coral L microparticles, collagen/coral S scaffolds and collagen/coral L scaffolds. (**b**) FTIR spectroscopy illustrating the absorbance peaks of groups at a wavelength of 850 cm^−1^. Peak areas were calculated for the different groups from a range of 840 to 865 cm^−1^.

In order to determine the crystalline structure of the materials, XRD was performed on coral microparticles and collagen/coral scaffolds, both of coral size L. XRD determined coral microparticles to be composed of calcium carbonate, which was primarily aragonite but also contained traces of calcite (Figure 2a, Supplementary Figure S1). Following the incorporation of coral microparticles into a collagen-based slurry, in which acetic acid was utilised as a solvent, the resultant freeze-dried collagen/coral scaffolds were determined to have a calcium ethanoate crystalline structure (Figure 2b). Collagen only scaffolds were not observed to have a crystalline structure (data not shown). To assess the influence of microparticle size on the rate of conversion from calcium carbonate to calcium ethanoate during the scaffold fabrication process, FTIR spectroscopy was performed on collagen/coral S and collagen/coral L scaffolds. FTIR spectroscopy demonstrated a large absorbance peak in coral microparticles at a wavelength of 850 cm^−1^ (corresponding to the presence of calcium carbonate) which was greatly reduced in both collagen/coral S and collagen/coral L scaffolds, indicating the conversion from calcium carbonate to calcium ethanoate (Figure 3a,b). To examine this effect further, peak areas were calculated for coral microparticles, collagen/coral S scaffolds and collagen/coral L scaffolds with results demonstrating similarly high rates of conversion, irrespective of the coral microparticle size used in the scaffold (Table 1). 

### 2.2. Architectural Properties of Collagen/Coral Scaffolds

**Figure 4:**
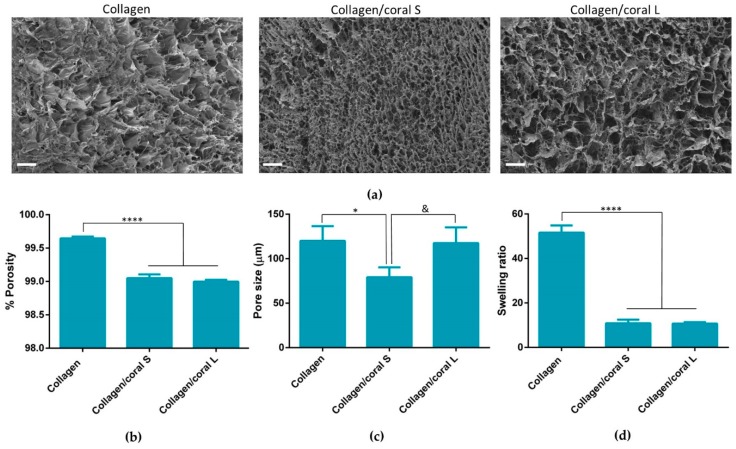
(**a**) Scanning electron microscopy images of collagen, collagen/coral S and collagen/coral L scaffolds. Scale bar–100 µm. Magnification–100×. (**b**) % Porosity of scaffolds (*n* = 4). (**c**) Pore size of scaffolds (*n* = 3). (**d**) Swelling ratio of scaffolds (*n* = 4). Significance; & *p* < 0.1, * *p* < 0.05, **** *p* < 0.0001.

Scanning electron microscopy demonstrated the presence of porous, interconnected networks in all scaffolds with collagen/coral S scaffolds appearing to contain smaller pore sizes when compared to collagen/coral L scaffolds and collagen only scaffolds (Figure 4a). The porosity of collagen only scaffolds (99.65 ± 0.03%) were found to be significantly higher compared to both collagen/coral S and collagen/coral L scaffolds (*p* < 0.0001) (Figure 4b). No significant differences in porosity were observed between collagen/coral S (99.05 ± 0.06%) and collagen/coral L (99 ± 0.03%). Differences in pore sizes were confirmed by histology, with a significant decrease in pore size observed in collagen/coral S scaffolds (79.13 ± 11.17 µm) compared to collagen scaffolds (120.1 ± 16.55 µm; *p* = 0.0397) and a trend towards a significant decrease observed in collagen/coral S scaffolds compared to collagen/coral L scaffolds (117.5 ± 17.69 µm; *p* = 0.0509) (Figure 4c). The swelling ratio of collagen scaffolds was found to be significantly greater when compared to both collagen/coral S and collagen/coral L scaffolds (*p* < 0.0001) (Figure 4d).

### 2.3. Mechanical Properties of Collagen/Coral Scaffolds

**Figure 5:**
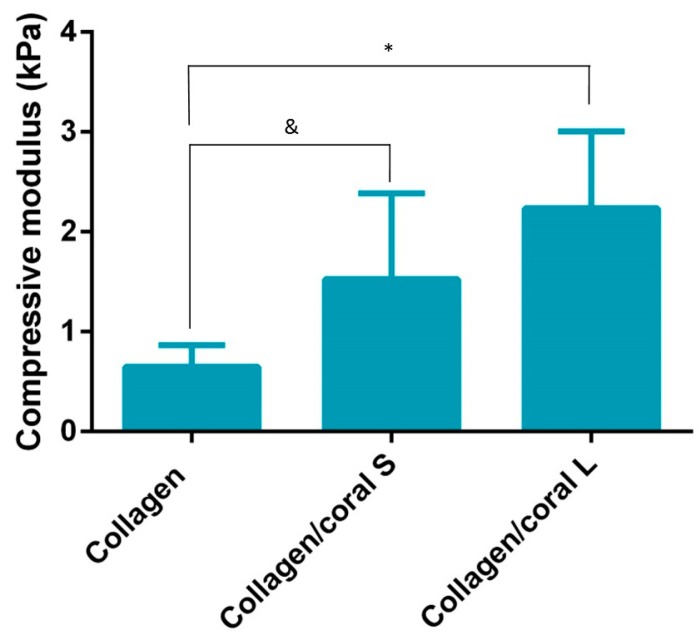
Compressive modulus of scaffolds (*n* = 6). Significance; & *p* < 0.1, * *p* < 0.05.

Prior to their mechanical and biological evaluation, scaffolds were cross-linked in order to sterilise the materials and enhance their compressive properties [24]. Collagen/coral L scaffolds demonstrated a significantly higher compressive modulus when compared to collagen only scaffolds (2.24 ± 0.77 kPa vs. 0.65 ± 0.22 kPa; *p* = 0.0028) whilst collagen/coral S scaffolds demonstrated a trend towards a significant increase in compressive moduli (1.53 ± 0.86 kPa; *p* = 0.0962) when compared to collagen scaffolds (Figure 5). No significant differences in compressive modulus were observed between collagen/coral S and collagen/coral L scaffolds.

### 2.4. Osteogenesis of Human MSCs Seeded on Scaffolds and in 2D Insert Culture

**Figure 6:**
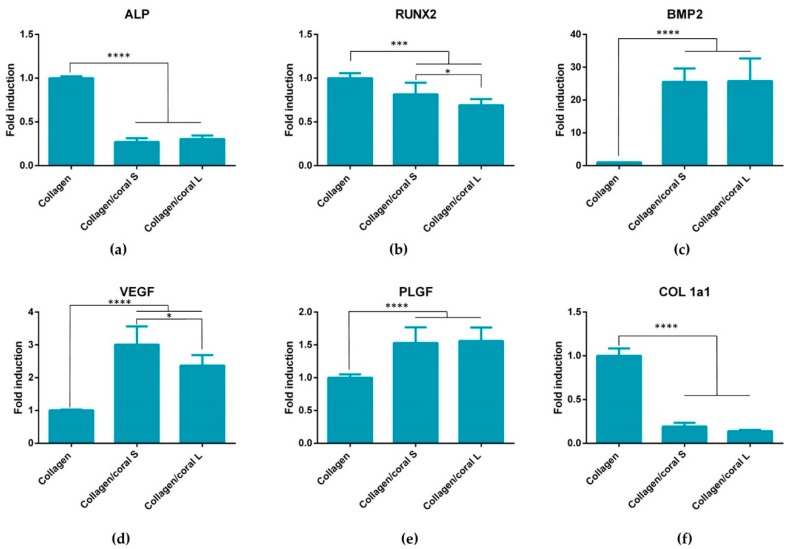
qPCR of mesenchymal stromal cell (MSC)-seeded scaffolds at day 7 assessing genes involved in the osteogenic and angiogenic processes (**a**) Alkaline phosphatase (ALP). (**b**) Runt-related transcription factor 2 (RUNX2). (**c**) Bone morphogenetic protein 2 (BMP2). (**d**) Vascular endothelial growth factor (VEGF). (**e**) Placental growth factor (PLGF). (**f**) collagen type I (COL 1a1). Data represents 2 donors with *n* = 5 scaffolds per group per donor. Significance; * *p* < 0.05, *** *p* < 0.001, **** *p* < 0.0001.

In order to evaluate their osteogenic capacity, collagen/coral scaffolds were seeded with human MSCs and maintained in an expansion medium for one day to allow attachment. Thereafter scaffolds were maintained in an osteogenic medium for the duration of the experiment (28 days total). Quantitative real time polymerase chain reaction (qPCR) was performed at day 7 to assess an array of genes involved in the osteogenic and angiogenic processes including alkaline phosphatase (ALP), runt-related transcription factor 2 (RUNX2), bone morphogenetic protein 2 (BMP2), vascular endothelial growth factor (VEGF), placental growth factor (PLGF) and collagen type I (COL 1a1). qPCR demonstrated a significant reduction in ALP gene expression in both collagen/coral S and collagen/coral L scaffolds compared to collagen only controls (*p* < 0.0001) (Figure 6a). The expression of RUNX was also down-regulated in both collagen/coral S and collagen/coral L scaffolds compared to collagen only controls (*p* < 0.001) (Figure 6b). Interestingly, however, the expression of BMP2 was determined to be profoundly up-regulated (25× increase) in collagen/coral S and collagen/coral L scaffolds when compared to controls (*p* < 0.0001) (Figure 6c). The expression of VEGF and PLGF genes were also significantly up-regulated in collagen/coral S and collagen/coral L groups, whilst COL Ia1 was shown to be down-regulated, when compared with controls (*p* < 0.0001) (Figure 6d–f). When comparing the two collagen/coral groups directly, collagen/coral S scaffolds demonstrated significant up-regulations in the expression of RUNX2 and VEGF compared collagen/coral L scaffolds (*p* < 0.05) (Figure 6b,d).

**Figure 7:**
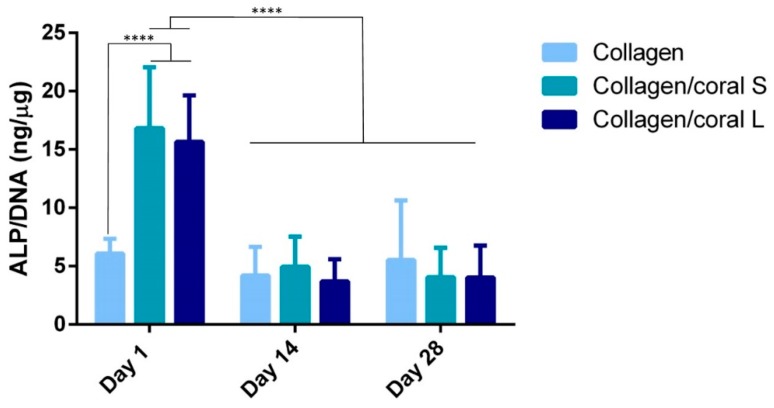
ALP/DNA of MSC-seeded scaffolds over 28 days in culture. Data represents 2 donors with *n* = 3–4 scaffolds per group per donor. Significance; **** *p* < 0.0001.

Having assessed the effect of collagen/coral scaffolds on the osteogenic differentiation of MSCs at the gene level, we next evaluated their expression at the protein level by firstly examining alkaline phosphatase (ALP) activity, an early marker of osteogenic differentiation. ALP/DNA was determined to be significantly enhanced at day 1 in both collagen/coral S (16.83 ± 5.23 ng/µg) and collagen/coral L (15.67 ± 3.97 ng/µg) scaffolds when compared to collagen only scaffolds (6.07 ± 1.27 ng/µg; *p* < 0.0001) (Figure 7). No significant differences in ALP/DNA were observed in collagen only scaffolds at days 1, 14 and 28, whereas ALP/DNA was significantly higher in both collagen/coral S and collagen/coral L groups at day 1 compared to days 14 and 28 (*p* < 0.0001).

**Figure 8:**
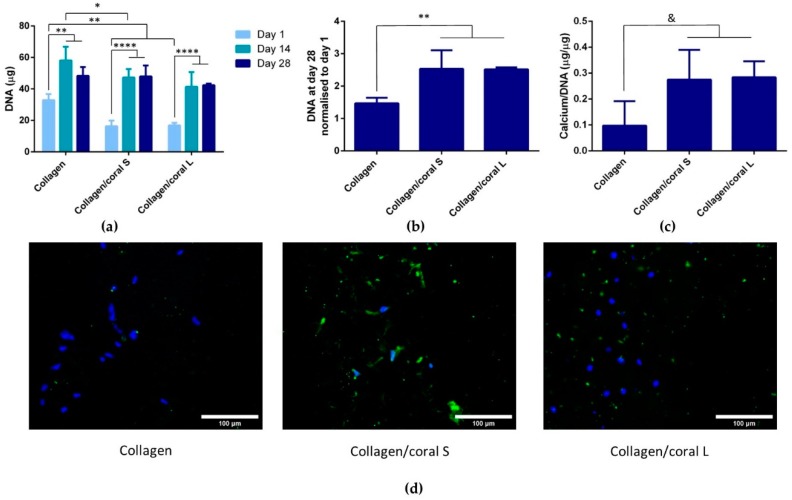
(**a**) DNA content of MSC-seeded scaffolds. *n* = 4 scaffolds per group. (**b**) DNA content of scaffolds at day 28 normalised to day 1. *n* = 4 scaffolds per group. (**c**) Calcium/DNA of MSC-seeded scaffolds at day 28. *n* = 3–4 scaffolds per group. (**d**) Indirect immunofluorescent staining for CaSR in MSC-seeded scaffolds at day 28. Magnification–20×. Images are representative of n=3 scaffolds per group. Data represents 1 donor. Significance; & *p* < 0.1, * *p* < 0.05, ** *p* < 0.01, **** *p* < 0.0001.

**Figure 9:**
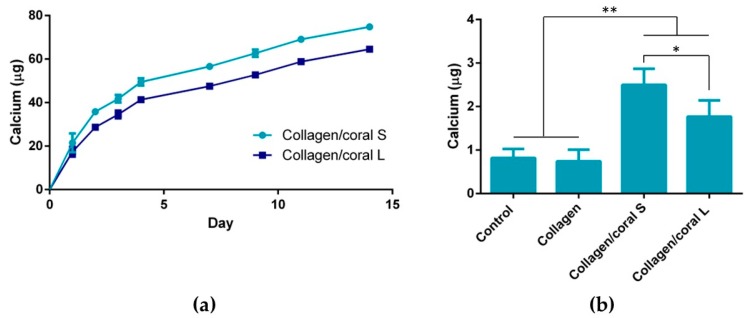
(**a**) Calcium measured in the media of collagen/coral S and collagen/coral L scaffolds over 14 days (*n* = 5). (**b**) Calcium accumulation of MSCs seeded in 2D and cultured either with no scaffolds present (control) or in the presence of collagen scaffolds, collagen/coral S scaffolds or collagen/coral L scaffolds. Data represents 1 donors with *n* = 4 samples per group. Significance; * *p* < 0.05, ** *p* < 0.01.

We next assessed the proliferation and calcium accumulation of MSC-seeded scaffolds after 28 days of osteogenic culture. At day 1, the DNA content of collagen scaffolds was found to be significantly higher when compared to both collagen/coral scaffolds (*p* < 0.01) (Figure 8a). The DNA content of all groups at days 14 and 28 were demonstrated to be significantly higher compared to their respective contents at day 1 (*p* < 0.01). No significant differences in DNA content were observed between groups at day 28. When normalised to their respective DNA contents at day 1, both collagen/coral S and collagen/coral L scaffolds were shown to significantly increase the proliferation of MSCs over collagen only scaffolds (*p* < 0.01) (Figure 8b). When compared to collagen only scaffolds at day 28, trends towards a significant increase in calcium/DNA content were observed in both collagen/coral S (*p* = 0.0825) and collagen/coral L (*p* = 0.0692) scaffolds (Figure 8c). Indirect immunoflourescence performed on MSC-seeded scaffolds at day 28 demonstrated intense staining for calcium sensing receptor (CaSR) in collagen/coral S groups with less intense, but still noticeable staining observed in collagen/coral L groups, whilst negligible levels of staining were observed in collagen only groups (Figure 8d). In order to determine whether enhancements in the osteogenesis of MSCs occurred as a result of soluble calcium ions delivered from the collagen/coral scaffolds, MSCs were seeded in 2D and cultured in the presence of scaffolds which were placed inside porous inserts so as to facilitate the transfer of soluble factors whilst ensuring no direct cell-scaffold contact. Calcium release assays performed in parallel demonstrated significantly increased calcium content in the release media of collagen/coral S scaffolds as compared to collagen/coral L scaffolds at day 1 (21.5 ± 4.36 µg vs. 17.4 ± 2.26 µg; *p* = 0.0024) through to day 14 (78.81 ± 1.48 µg vs. 64.55 ± 0.4 µg; *p* < 0.0001) (Figure 9a). At day 14, the calcium accumulation of MSCs seeded in 2D was evaluated. MSCs cultured in the presence of both collagen/coral S and collagen/L scaffolds accumulated significantly more calcium when compared to MSCs cultured in the presence of collagen only scaffolds and control MSCs cultured in scaffold-free conditions (*p* < 0.01) (Figure 9b). On comparing the two collagen/coral scaffold groups, MSCs cultured in the presence of collagen/coral S scaffolds were found to accumulate significantly more calcium when compared to MSCs cultured in the presence of collagen/coral L scaffolds (2.5 ± 0.37 µg vs. 1.77 ± 0.37 µg; *p* = 0.0276).

## 3. Discussion

This work sought to assess the effect of incorporating marine coral microparticles of different sizes on the material, mechanical and osteogenic properties of collagen-based scaffolds. To that end, cryomilling was utilised to obtain coral microparticles of mean diameters 14 µm and 64 µm (S and L), respectively. The coral used in the study was demonstrated to be composed of calcium carbonate whilst collagen/coral scaffolds fabricated by freeze-drying were determined to have a crystalline calcium ethanoate structure. The incorporation of coral microparticles was found to alter the architecture of collagen-based scaffolds, with lower porosities observed in both collagen/coral scaffolds, and smaller pore sizes observed in collagen/coral S scaffolds. Coral incorporation was also demonstrated to increase the mechanical properties of collagen-based scaffolds, with a more profound increase in compressive stiffness observed in the collagen/coral L group. Most importantly, the addition of coral microparticles of both sizes to collagen-based scaffolds was found to greatly enhance the expression of BMP2 at the gene level, and ALP activity and calcium accumulation at the protein level, of human MSCs. This indicates that coral microparticles can be leveraged to promote a robust osteogenic phenotype in human MSCs with this induction likely occurring due to calcium ion signalling resulting from their crystalline calcium ethanoate structure. Taken together, the improved mechanical and osteogenic properties of collagen/coral scaffolds illustrate their potential use as novel grafts for orthopaedic and tissue engineering applications such as bone regeneration.

Coral are marine invertebrates which have been proposed to offer the potential to act as natural bioceramic scaffolds for bone repair. The XRD analysis carried out in this study confirmed the coral skeleton to be composed of calcium carbonate, primarily aragonite but also containing traces of calcite, which tallies well with other studies in the field that have examined the mineral composition of marine corals [25,26]. Upon the incorporation of coral microparticles into a collagen-based slurry, the subsequent freeze-dried collagen/coral scaffold was shown to have a crystalline calcium ethanoate structure. Calcium ethanoate is a calcium salt of acetic acid which traditionally has been produced by soaking carbonate rocks, such as limestone, in vinegar. As the collagen used in the fabricated scaffolds was initially blended in acetic acid to form a slurry, the addition of coral microparticles to the slurry was determined to follow that conversion process from calcium carbonate to the more soluble calcium ethanoate. In order to assess whether the size of the coral microparticles influenced the degree of conversion from carbonate to ethanoate, we utilised FTIR spectroscopy to examine the peak observed at the wavelength of 850 cm^−1^ which is used to identify the presence of calcium carbonate [27]. That this peak was almost completely obliterated in collagen/coral scaffolds irrespective of the microparticle size used in their fabrication would appear to suggest a very high conversion rate occurring during the blending/freeze-drying process. 

An advantage of utilising collagen-based scaffolds for tissue engineering applications is their highly porous nature, which allows for the rapid infiltration of cells into central regions of the scaffold whilst facilitating the transfer of nutrients and removal of waste products. This porosity is a result of the freeze-drying process whereby the ice crystals that form within the slurry during freezing are sublimed through the addition of heat at a reduced pressure, thereby ensuring that the solid ice crystals are transformed directly into a vapour leaving in their stead a highly porous interconnected network [28]. In this study, the incorporation of coral microparticles was found to reduce the overall porosity of the scaffold, although that porosity still remained extremely high (≥99%). Another architectural characteristic which influences cellular activity is the size of the pores developed within the scaffold. Pore size is related to the remodelling of the ice crystal structure below freezing temperature and can be regulated by altering the final freezing temperature, freezing rate and composition of the slurry [29]. Interestingly, it was upon assessment of the pore sizes which developed within collagen/coral scaffolds that a notable difference was observed depending on the size of the microparticle used, with collagen/coral S scaffolds demonstrating average pore sizes of 79.13 µm whilst collagen/coral M scaffolds showed pore sizes similar to those of collagen only controls at 117.5 µm. Although the overall concentrations of coral microparticles used in the scaffolds were the same, other factors may have influenced the final pore sizes of the scaffolds. For example, studies have shown that differences in pH (from 2.8 to 2.5) can result in two-fold increases in the pore size of collagen sponges [30], and it may be that such variances are introduced into the scaffold fabrication system due to the on-going calcium carbonate/calcium ethanoate conversion. Nevertheless, the pore sizes of the collagen/coral scaffolds described here are in the range of those previously deemed suitable by our lab for bone tissue engineering applications [16].

The mechanical properties of scaffolds for applications such as bone regeneration is an important consideration as the biomaterial should be of sufficient strength so as to facilitate mechanical functionality in vivo and to ensure ease-of-handling for a surgeon performing the implantation. Processes that can be leveraged to enhance the compressive properties of collagen-based scaffolds include physical and chemical crosslinking actions such as the dehydrothermal and carbodiimide treatments used herein which form crosslinks between the carboxyl and amino acid groups of varying residues in the collagen. The mechanical properties of collagen-based scaffolds can be further increased through the incorporation of additional compounds such as HA [20] and chitosan [31]. The addition of marine coral was also found to enhance the compressive properties of collagen-based scaffolds, with a more profound improvement in stiffness observed in the collagen/coral L group.

Another key attribute of any graft designed for bone tissue engineering is the capacity of that graft to promote osteogenesis of progenitor cells, and this applies for a scaffold whether it is seeded with cells and primed in vitro, or implanted into the body cell-free with the aim of facilitating infiltration of host cells. Herein, we utilised mesenchymal stromal cells, isolated from the bone marrow of two human donors, as a model through which we could assess the potential of collagen/coral scaffolds to act as grafts for promoting bone regeneration. When examined at the gene level, collagen/coral scaffolds were shown to massively up-regulate the expression of BMP2, reaching levels 25x higher than collagen only controls. BMP2 is a member of the transforming growth factor superfamily and plays a crucial role in development of bone during endochondral skeletogenesis [32]. BMP2 has been commercialised in the form of a recombinant protein which initially was shown to have tremendous effects on bone regeneration, although recent evidence has come to light which has demonstrated significant adverse side effects attributed to the supraphysiological levels of protein applied [33,34,35]. However, a scaffold which can up-regulate BMP2 expression at the gene level in the absence of any exogenously applied BMP2 could potentially be leveraged as a safer alternative and thus a more attractive proposition from a regulatory perspective. Other genes up-regulated in collagen/coral groups included VEGF, a potent promoter of angiogenesis, and PLGF, mechanosensitive gene which has been demonstrated by our group to impart a dose-dependent response on angiogenesis and osteogenesis, with higher doses promoting angiogenesis whilst lower doses promote osteogenesis [36]. Genes determined to have been down-regulated in the collagen/coral groups included ALP, although this effect can possibly be explained by the large increase in ALP activity observed at the protein level in collagen/coral scaffolds as illustrated in Figure 7, which occurred after just one day. It is therefore likely that certain genes involved in the osteogenic cascade, such as ALP and RUNX2, are up-regulated at the protein level quite rapidly and that the time point used for the assessment of gene expression herein (day 7) was too late. Interestingly, the osteogenic factors β-glycerophosphate, dexamethasone and ascorbic acid were only added to the culture media after day 1, which suggests that even in the absence of these potent biochemical factors, collagen/coral scaffolds have the capacity to promote a more robust osteogenic phenotype in human MSCs. The DNA contents of collagen/coral scaffolds at day 1 were found to be lower than in collagen only scaffolds indicating that, to a certain extent, the incorporation of coral microparticles into collagen-based scaffolds inhibits initial cellular attachment. This is possibly explained by the greater capacity of collagen scaffolds to swell upon hydration, as an increased swelling ratio is typically associated with improved cellular adhesion which is initially most prominent along the periphery of the scaffold [16]. Thereafter, however, MSCs seeded on collagen/coral scaffolds proliferated throughout the scaffold to a greater degree. This increased rate of proliferation may be caused by the enhanced structural integrity introduced in polymer/ceramic composite scaffolds which results in better maintenance of the interconnected pore structure and increased permeability [20].

A unique characteristic of the collagen/coral scaffolds developed in this study is their calcium ethanoate crystalline structure, the high solubility of which results in delivery of calcium ions to surrounding cells. After 28 days of culture in osteogenic medium, MSC-seeded collagen/coral scaffolds both demonstrated strong trends towards significant increases in calcium accumulation when compared to collagen only scaffolds. That the trends observed did not become significant may be a limitation of the biochemical assay used to assess calcium accumulation, as it requires digestion of the entire cell-seeded scaffold and subsequently subtracting from its accumulation the values obtained from digested cell-free controls which are inherently rich in calcium. Interestingly, however, MSC-seeded collagen/coral scaffolds both demonstrated positive staining for CaSR. CaSR is G protein-coupled receptor which binds to extracellular calcium ions [37,38] and has been demonstrated to promote osteogenic differentiation of MSCs in vitro [39,40] and to determine the route through which bone formation occurs in vivo, with hyperstimulation of CaSR being shown to inhibit chondrogenic differentiation thus directing MSCs down an intramembranous ossification pathway [41]. Interestingly, previous studies have shown that extracellular calcium ions, delivered from calcium phosphate glass/polylactic acid scaffolds and acting through CaSR, also elicits a pro-angiogenic effect in endothelial progenitor cells [42]. It may be, therefore, that the up-regulation of angiogenic genes observed in collagen/coral scaffolds is also occurring through this pathway, a process could potentially be leveraged as an additional therapeutic tool in promoting bone regeneration. In order to explore further the mechanisms through which enhanced osteogenesis occurs in collagen/coral scaffolds, a scaffold insert model was used to evaluate the effect of soluble calcium ions on underlying cells seeded in 2D. MSCs receiving soluble factors for collagen/coral scaffolds underwent robust osteogenesis resulting in significantly more calcium accumulation than MSCs in control groups, indicating that calcium ion signalling is the mechanism through which enhanced osteogenesis is occurring in these scaffolds. It should be noted, however, that the environment experienced by cells alters significantly when switched from a 3D to a 2D culture set-up, with changes in substrate stiffness [43], oxygen availability [44] and nutrient diffusion [45] all potentially playing a role in ECM deposition.

On examining the influence of microparticle size on the material, mechanical and biological characteristics of collagen/coral scaffolds, it can be determined that only subtle differences arose when utilising either 14 µm or 64 µm particle sizes within the scaffold. It is likely that the conversion from calcium carbonate to calcium ethanoate which occurs upon the addition of the coral microparticles to the collagen/acetic acid slurry results in generation of scaffolds both containing more soluble forms of calcium and thus more similar in their composition. Nevertheless, some small differences were observed, such as the improved mechanical properties of scaffolds fabricated using 64 µm particles and the enhanced calcium release kinetics of scaffolds fabricated using 14 µm particles, which would suggest that larger particles sizes lend themselves to the development of scaffolds with greater compressive stiffnesses whilst smaller particles result in scaffolds with more effective calcium ion diffusion properties. Ultimately, however, it can be concluded that irrespective of their size, the incorporation of marine coral microparticles improves the properties of collagen-based scaffolds for use in orthopaedic applications such as bone repair, and raises the possibility of further optimisation of the biomaterial, such as through their functionalisation with growth factors and genes involved in the regenerative process [46,47]. Alternatively, the scaffolds could potentially be implanted gene/growth factor-free which may reduce the likelihood of any adverse side effects occurring. Future evaluation of these scaffolds in relevant animal models would give a further indication of their suitability for use as grafts for promoting bone regeneration.

## 4. Materials and Methods 

### 4.1. Generation and Size Measurement of Coral Microparticles

Marine coral was donated by Zoan Biomed Ltd. (Galway, Ireland). The coral was first ground using a pestle and mortar and then cryomilled in 3 cycles of 1 min each, with a 1 min rest in between, using a Spex Sample Prep 6775 Freezer/Mill. Thereafter, the cryomilled particles were passed through a 100 µm sieve and then a 45 µm sieve (both Fisher Scientific, Loughborough, UK). Particles which had passed through the 45 µm sieve were collected and assigned as S and particles which had passed through the 100 µm sieve but not the 45 µm sieve were collected and assigned as L. The size profiles of the S and L coral particles were determined by dynamic light scattering using a Mastersizer 2000 (Malvern Panalytical Ltd., Malvern, UK). Briefly, the dispersant chamber was filled with ethanol and coral particles were added directly to the chamber under stirring at 1260 rpm until the laser obscuration value passed 10%. The refractive index of ethanol and coral were taken to be 1.36 and 1.545, [48,49]. The resultant particle size determined is reported as mean volume weighted diameter.

### 4.2. Fabrication of Collagen and Collagen/Coral Scaffolds

Two grams of type I collagen from isolated from bovine tendon (Collagen Solutions, Glasgow, UK) was added to 360 mL of 0.5 M glacial acetic acid (Sigma Aldrich, Wicklow, Ireland) and blended at 13,000 rpm for 90 min at a temperature of 4 °C using an overhead blender (Ultra Turrax T18 Overhead Blender, IKA Staufen, Germany). In order to form a collagen only slurry, 10 mL of 0.5 M acetic acid was added to 90 mL of slurry and blended for a further 30 min to give a final concentration of 0.5% w/v collagen. In order to form collagen/coral slurries, 1 g of each coral microparticle size (S or L) in 10 mL of stock acetic acid was separately incorporated into 90 mL of slurry to give two separate collagen/coral slurries which were blended for a further 30 min to give final concentrations of 0.5% w/v collagen, 200% wt. coral for each coral microparticle size. Slurries were degassed under a vacuum, poured into stainless steel moulds, and freeze-dried (Advantage EL, Vis-Tir Co., Gardiner NY) to a final temperature of −40 °C according to a previously optimised protocol to form porous 3D scaffolds of height ≈ 4 mm (Supplementary Figure S2). Cylindrical scaffolds of diameter 10 mm were used to characterise the material, architectural and mechanical properties of the scaffolds. Cylindrical scaffolds of diameter 8 mm were used to evaluate the osteogenic capacities of the scaffolds.

### 4.3. XRD and FTIR of Coral Microparticles and Collagen/Coral Scaffolds

XRD was utilised to determine the mineral composition of coral and collagen/coral scaffolds. One hundred milligrams of coral microparticles and 100 mg of collagen/coral scaffolds were separately assessed using a Bruker D8 Advance Diffractometer (Malvern Panalytical Ltd., Malvern, UK) using Cu Kα1, Kα2 radiation. A 20 µm nickel filter placed prior to the Lynxeye solid state detector was used to filter ≈99% of unwanted radiation. A step size of 0.01° two theta was utilised, and data was collected from 5 to 60° two theta. A fixed divergence slit of 0.3° was used throughout. A Bruker Alpha Fourier Transform Infra-Red spectrometer fitted with an ATR sampling accessory was used to measure the IR spectra of coral microparticles and collagen/coral scaffolds. The samples were placed directly on the ATR crystal and spectra were recorded in reflection mode. Next, 512 scans at 4 cm^−1^ resolution were integrated to obtain the spectra. Peak areas were calculated by determining the integral of the curves from 840 to 865 cm^−1^.

### 4.4. Characterisation of the Architecture of Collagen/Coral Scaffolds

The architecture of collagen/coral scaffolds was visualised using scanning electron microscopy. Scaffolds were mounted onto metallic studs using carbon cement prior to being sputtered with a gold/palladium alloy and imaged using a Zeiss Ultra Plus electron microscope (ZEISS, Jena, Germany). The porosity of the scaffolds was calculated according to the following equation

$$\%\;Porosity=\;\frac{\rho \;scaffold}{\rho \;material}\;\times \;100$$

where *ϱ* *material* is the density of the material from which the scaffold is fabricated and *ϱ* *scaffold* is the apparent density of the scaffolds measured by dividing the weight by the volume of the scaffold. The pore size of scaffolds was quantified using a histological technique as previously described [50]. Briefly, Ø 10 mm scaffold samples were embedded in JB-4^®^ glycolmethacrylate (Polysciences Europe, Eppelheim, Germany) and serially sectioned at 10 µm, both longitudinally and transversely, using a microtome (Leica RM 2255, Leica, Wetzlar, Germany) and stained with toluidine blue. A pore topology analyser developed using MATLAB (MathWorks Inc, Natick, MA, USA) was used to quantify the average pore size of each scaffold. The swelling ratio of scaffolds were determined by hydrating the scaffolds in a graded series of ethanol and maintaining the scaffolds in distilled water for 24 h at room temperature. Swelling ratio was calculated according to the following equation

$$Swelling\;ratio=\;\frac{w-d}{d}$$

where *d* is the dry weight of the scaffold and *w* is the wet weight of the scaffold.

### 4.5. Crosslinking of Scaffolds

Prior their mechanical and biological evaluation, scaffolds were cross-linked in order to enhance their mechanical properties and sterilise the scaffolds. Scaffolds were firstly dehydrothermally cross-linked by placing them in a vacuum oven (Vacucell 22; MMM Medcenter, Munich, Germany) at 105 °C and 0.05 bar for 24 h. As well as sterilising the scaffolds, the elevated temperature causes condensation reactions between the carboxyl groups of aspirate or glutamate residues and the amino acids of lysine or hydroxylysine, resulting in the formation of intermolecular cross-links. Thereafter, scaffolds were chemically cross-linked using a mixture of 6 mM *N*-(3-Dimethylaminopropyl)-*N*’-ethylcarbodiimide hydrochloride and 5 mM *N*-Hydroxysuccinimide in ethanol, which forms zero-length cross-links in collagen between the carboxyl and amino groups of varying residues and increases the mechanical properties of the scaffold [24,51].

### 4.6. Mechanical Testing of Scaffolds

The mechanical properties of scaffolds were assessed using uni-axial, unconfined compressions tests performed in a saline (0.9%) bath between impermeable platens by a mechanical testing machine (Z050, Zwick-Roell, Ulm, Germany) fitted with a 5 N load cell. Prior to testing, Ø 10 mm scaffolds were hydrated in phosphate buffered saline (PBS) for 1 h. Scaffolds were compressed to a final strain of 10% at a strain rate of 10%/min. The stress experienced by each scaffold was calculated by dividing the applied force by the cross-sectional area of the scaffold and the resulting stress–strain curve was plotted. The compressive modulus of each scaffold was calculated by determining the slope of a linear fit to the stress strain curve between 2% and 5% strain.

### 4.7. Osteogenic Differentiation of Human MSCs Cultured on Scaffolds and in 2D

MSCs were isolated from donated human tissue after obtaining permission for their use in research applications by informed consent or legal authorization. Detailed procedures on the isolation and characterisation of human MSCs can be found in [52]. Briefly, human bone marrow derived MSCs were isolated from the iliac crest of 20–30 year old adults (Lonza Biologics PLC, Slough, UK) and seeded at a density of 1 × 10^6^ MSCs per T175 flask in an expansion medium consisting of Dulbecco’s modified Eagle’s medium (DMEM) supplemented with 10% foetal bovine serum (Labtech, East Sussex, UK) and 1% penicillin/streptomycin (Sigma Aldrich, Wicklow, Ireland) and expanded to passages 4–5. Cylindrical scaffolds of dimensions Ø 8 × 4 mm were seeded with 400,000 MSCs (2000 MSCs per mm^3^ of scaffold) and maintained in expansion medium for 24 h to allow for cells to adhere. Thereafter, scaffolds were transferred to an osteogenic medium consisting of DMEM supplemented with 10% foetal bovine serum, 1% penicillin/streptomycin, 10 mM β-glycerophosphate, 50 µm ascorbic 2-phosphate and 100 nM dexamethasone (all Sigma-Aldrich, Wicklow, Ireland). The media was replaced twice weekly up to an experimental end point of 28 days. At time points of day 1, 14 and 28, scaffolds were removed from their culture media, washed in PBS, frozen, and stored at −80 °C for biochemical analysis. MSCs were also plated in 2D in 24 well plates, at a density of 10,000 MSCs/well, containing scaffolds placed in 0.4 µm hanging cell culture inserts (Millicell) to allow for the transportation of soluble factors from the scaffold whilst prevent any MSC-scaffold contact. MSCs were maintained in expansion medium for 24 h to adhere and thereafter were cultured in osteogenic medium up to a time point of 14 days with media changes occurring twice weekly.

### 4.8. qPCR Analysis of Osteogenic and Angiogenic Gene Expressin

mRNA was isolated from 3 × 10^5^ MSCs cultured for seven days in osteogenic medium on collagen, collagen/coral S and collagen/coral L scaffolds. Scaffolds were rinsed twice with PBS and 500 µL of Qiazol lysis buffer (Qiagen, UK) was added to each scaffold prior to freezing and storing at −80 °C. An RNeasy Minikit (Qiagen, UK) was then utilised according to the manufacturer’s instructions. Briefly, two-step reverse transcription and real-time PCR were performed using Quantitect Reverse Transcription Kits (Qiagen, UK) and Sensimix SYBR low Rox PCR Kits (Medical Supply Company, IE), respectively, loading 2.5 ng of cDNA per reaction. Primer amplification efficiency was compatible with the comparative ΔΔCt method used for expression of the results. The fold induction index was normalised by the housekeeping expression of each sample to compare the genetic expression of cells cultured in collagen/coral S or collagen/coral L scaffolds against the expression in collagen scaffolds. The primers used for the real time PCR were: alkaline phosphatase (ALP), (Qiagen, reference QT00012957); runt-related transcription factor 2 (RUNX2), (Qiagen, reference QT00020517); placental growth factor (PLGF), (Qiagen, reference QT00030688); vascular endothelial growth factor (VEGF), (Qiagen, reference QT01010184); bone morphogenetic protein 2 (BMP2), (Qiagen, reference QT00012544); and house-keeping 18S (Qiagen, reference QT00199367). The PCR was initiated with an activation step of 15 min at 95 °C, followed by 40 cycles of denaturation (15 s, 94 °C), annealing (30 s, 55 °C) and extension (30 s, 72 °C), followed by the melting curve as recommended by the manufacturer, in an Eppendorf^®^ Mastercycler^®^ ep realplex 4.

### 4.9. Indirect Immunofluorescent Staining for CaSR

At day 28 of osteogenic culture, MSC-seeded scaffolds and MSC-free scaffold controls were fixed in formalin (Sigma-Aldrich, Wicklow, Ireland) overnight, dehydrated through a graded series of ethanols and xylenes, and embedded in paraffin. Paraffin blocks were sectioned at 5 µm using a microtome (Leica, Wetzlar, Germany) and affixed to slides. Slides were deparaffinised, hydrated to 0.1% Tween 20 (Sigma-Aldrich, Wicklow, Ireland) in PBS and incubated in blocking buffer for 1 h. Thereafter, samples were washed in 0.1% Tween 20 in PBS and incubated in mouse calcium sensing receptor monoclonal antibody HL 1499 (ThermoFisher Scientific, Waltham, MA, USA; 1 mg/mL) at a dilution of 1:100 overnight at 4 °C. Samples were then washed and incubated in goat anti-mouse IgG alexa fluor plus 488 (ThermoFisher Scientific; 2 mg/mL) at a dilution of 1:250 for 1 h and counterstained with DAPI (1 mg/mL) at a dilution of 1:500 which stains nuclei blue and mounted with Fluoromount Aqueous Mounting Medium (Sigma Aldrich, Wicklow, Ireland) (Supplementary Figure S3). Images were taken using a Nikon Microscope Eclipse 90i with NIS Elements Software v3.06 (Nikon instruments Europe, Amstelveen, The Netherlands).

### 4.10. Biochemical Analysis of MSC Seeded on Scaffolds and in 2D

Upon thawing, samples were homogenised in 1 mL of lysis buffer consisting of PBS supplemented with 2% Triton-x-100 and centrifuged for 15 min at 10,000 G and 4 °C. DNA quantification was carried out using a Quant-iT™ PicoGreen^®^ dsDNA assay kit (Biosciences, Dublin, Ireland) with a Lambda DNA standard. Alkaline phosphatase activity was measured using a Sensolyte pNPP Alkaline Phosphatase assay kit (Cambridge Bioscience, Cambridge, UK) with a calf intestine alkaline phosphatase standard. The calcium content of scaffolds was measured by digesting samples in 1 mL of 0.5 M hydrochloric acid (HCL) and using a StanBio Calcium Liquicolour Kit (ThermoFisher Scientific, Waltham, MA, USA) according to the manufacturer’s instructions. The calcium content of cell-free scaffolds cultured in parallel was subtracted from the content of MSC-seeded scaffolds. The calcium content of MSCs seeded in 2D was measured by adding 1 mL of 0.5 M (HCL) to each well, using a cell scrapper to detach cells from the well, and performing the StanBio calcium assay as described above.

### 4.11. Calcium Release Assays

Scaffolds were placed in 2 mL of PBS which was collected and replaced with fresh PBS at 1, 2, 3, 4, 7, 9, 11 and 14 day time points. Calcium released into the PBS at these time points was calculated using the StanBio calcium assay kit.

### 4.12. Statistical Analysis

Statistical analyses were performed using Graphpad Prism 6.0 and, unless otherwise stated, one and two-way ANOVAs were performed followed by Tukey’s test to compare conditions. Significance was accepted at a level of *p* < 0.05. Results are presented as mean ± standard deviation from the mean.

## 5. Conclusions

Herein, we report the development and characterisation of a novel biomaterial scaffold for use as a graft in musculoskeletal tissue engineering applications. This composite biomaterial, generated by incorporating marine coral microparticles into a collagen-based scaffold, was shown to be highly porous and demonstrated enhanced mechanical properties when compared to collagen only scaffolds. Furthermore, the unique calcium ethanoate crystalline structure of the biomaterial allowed for the effective delivery of calcium ions which was shown to trigger the CaSR signalling pathway and promote robust osteogenesis of human MSCs. This novel scaffold, fabricated from entirely natural sources, therefore shows promise as graft which could potentially be leveraged as an alternative to both autografts and growth-factor loaded biomaterials for applications such as bone repair.

## Figures and Tables

**Table 1:** Areas calculated for the FTIR absorbance peaks occurring in the wavenumber region from 840 to 865 cm^−1^ as illustrated in Figure 3b.

	Coral L Microparticles	Collagen/Coral S Scaffolds	Collagen/Coral L Scaffolds
**Peak area**	4.525	0.026	0.021

## Supplementary Materials

The following are available online at https://www.mdpi.com/1660-3397/18/2/74/s1, Figure S1: XRD analysis of coral L microparticles. Blue dashed box indicates the region presented in Figure 2a. Control spectra were obtained from the International Centre for Diffraction Data (ICDD); Ca C O3 Aragonite—PDF 00-041-1475 (ICDD, 2019), Ca C O3 Calcite, syn—PDF 00-005-0586 (ICDD, 2019). CPS indicates counts per second; Figure S2. Macroscopic images of cylindrical scaffolds. Scale bar—10 mm. Figure S3. Indirect immunofluorescent staining for CaSR in MSC-seeded scaffolds at day 28. Green channel (top row) represents CaSR staining. Blue channel (bottom row) represents DAPI staining. Magnification—20x. Images are representative of n = 3 scaffolds per group. Data represents 1 donor.
