# Supplementary material for: The Incorporation of Marine Coral Microparticles into Collagen-Based Scaffolds Promotes Osteogenesis of Human Mesenchymal Stromal Cells via Calcium Ion Signalling
Source: Mar Drugs. 2020 Jan 23;18(2):74. doi: 10.3390/md18020074 (PMC7073845; doi:10.3390/md18020074)
Supplement: Supplementary file 1 [file marinedrugs-18-00074-s001.pdf]

Supplementary data

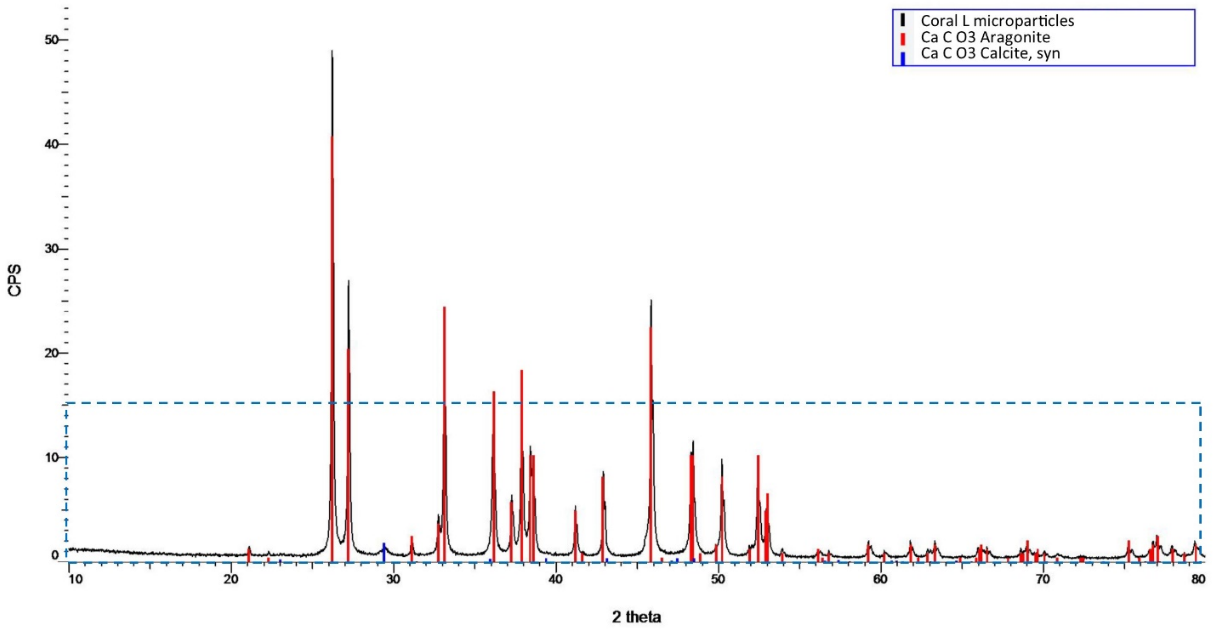

Supplementary Figure S1. XRD analysis of coral L microparticles. Blue dashed box indicates the region presented in Figure 2a. Control spectra were obtained from the International Centre for Diffraction Data (ICDD); Ca C O3 Aragonite – PDF 00-041-1475 (ICDD, 2019), Ca C O3 Calcite, syn – PDF 00-005-0586 (ICDD, 2019). CPS indicates counts per second.

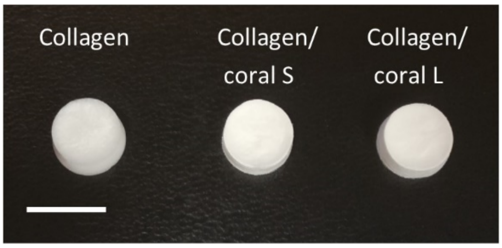

Supplementary Figure S2. Macroscopic images of cylindrical scaffolds. Scale bar – 10 mm.

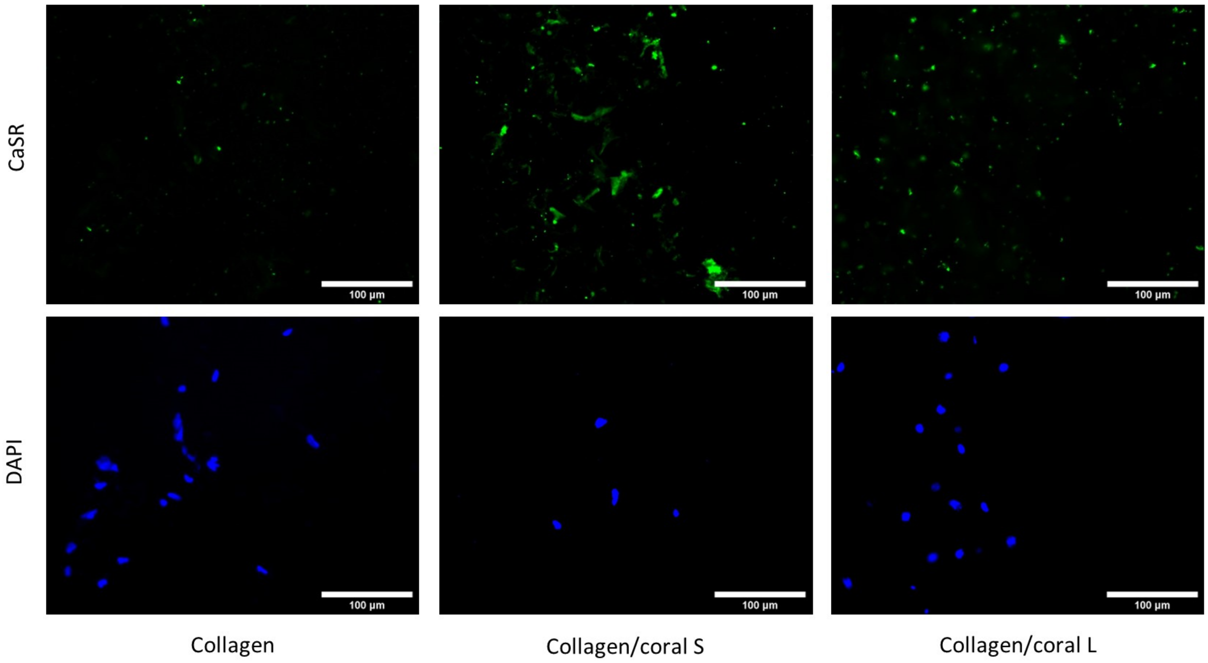

Supplementary Figure S3. Indirect immunofluorescent staining for CaSR in MSC-seeded scaffolds at day 28. Green channel (top row) represents CaSR staining. Blue channel (bottom row) represents DAPI staining. Magnification – 20x. Images are representative of n=3 scaffolds per group. Data represents 1 donor.
